# Letter to the Editors: Comments on “Clinical and Imaging Correlates of Medication Reduction in Globus Pallidus Stimulation for Parkinson's Disease”

**DOI:** 10.1002/mdc3.70126

**Published:** 2025-05-12

**Authors:** Luigi G. Remore, Filippo Cogiamanian, Ioannis U. Isaias, Marco Locatelli

**Affiliations:** ^1^ Neurosurgery Unit Fondazione IRCCS Ca' Granda Ospedale Maggiore Policlinico Milan Italy; ^2^ Department of Pathophysiology and Transplantation University of Milan Milan Italy; ^3^ Neuropathophysiology Unit Fondazione IRCCS Ca' Granda Ospedale Maggiore Policlinico Milan Italy; ^4^ Parkinson Institute of Milan, ASST G. Pini‐CTO Milan Italy; ^5^ University Hospital of Wuerzburg and Julius Maximilian University of Wuerzburg Wuerzburg Germany

We read with great interest the paper by Di Luca et al.^1^ After a VTA‐based analysis, the authors identified the anteromedial GPi as the stimulation site associated with supra‐threshold LEDD reduction in PD patients undergoing GPi‐DBS. No clinical or demographic variables significantly contributed to LEDD reduction in a multivariate logistic regression model; thus, the anatomic location was confirmed to be the only factor determining pharmacological variations in the analyzed cohort. The study has some limitations, mainly its retrospective nature, the relatively small number of patients included and the chosen threshold for LEDD reduction (ie, >30%) that was justified by literature evidence, although LEDD reduction after STN‐DBS is often much more pronounced in the clinical practice as well as patients’ expectations are. Nonetheless, we think that this study is important and challenges the two main actors involved in target selection: the neurosurgeon, who should not take for granted that only stimulation of the posteroventral GPi provides motor improvement; the neurologist, who should not refrain from offering GPi‐DBS to PD patients because of supposed inferior reduction in anti‐parkinsonian medications.

Interestingly, the results of this study contrast with a recent paper by Holland et al,^2^ who proposed the dorsolateral GPi as an ideal target. Specifically, stimulation of the dorsal GPi closer to GPe yielded inferior UPDRSIII ON/OFF scores, less ON dyskinesias and more pronounced LEDD reduction than GPi subcomponents distant from the medial medullary lamina (mml). Conversely, Di Luca et al^1^ reported sub‐threshold LEDD reduction in stimulation volumes close to the mml and cited existing evidence against GPe‐stimulation. We suspect that these opposing results may reflect different methodologies: Holland et al^2^ localized contacts with clinically available software, while Di Luca et al^1^ performed a VTA‐based probabilistic voxel analysis. Both methods have various limitations, specifically concerning the degree of anatomic reliability. Further clinical studies with larger cohorts and possibly randomized designs are needed to clarify which surgical target is the most beneficial between the dorsal and anteromedial GPi.

The paper by Di Luca et al^1^ has two potential clinically relevant implications. Firstly, more widespread adoption of GPi‐DBS would result, if the drug‐sparing effect of GPi‐stimulation was confirmed. Indeed, especially European neurologists tend to reserve GPi‐DBS for older and more dyskinetic PD patients, who would not tolerate abrupt drug reduction after STN‐DBS.^3^ Secondly, the LEDD‐reduction effect of GPi‐stimulation would benefit the “renaissance” of lesional procedures. Di Luca et al^1^ cited the LEDD‐sparing effect of more anteromedial targeting during classical pallidotomies, while a recent randomized trial showed promising results for MRI‐guided FUS pallidotomy.^4^ Although drug dosages were kept constant during the follow‐up period as required by the trial's protocol,^4^ an LEDD‐reducing effect might hasten the clinical application of FUS pallidotomy. However, concerns remain about the yet‐described cognitive sequelae after lesions of the limbic anteromedial GPi^5^ further underscored the different pathogenetic mechanisms underlying stimulation and lesioning.

In conclusion, we applaud the authors for their study. We believe their work will push neurologists to overcome strict dogma in target selection and neurosurgeons to tailor the target to patients’ characteristics.

## Author Roles

(1) Research project: A. Conception, B. Organization, C. Execution; (2) Statistical Analysis: A. Design, B. Execution, C. Review and Critique; (3) Manuscript Preparation: A. Writing of the first draft, B. Review and Critique.

L.G.R.: 1A, 1B, 1C, 3A.

F.C.: 3B.

I.U.I.: 3B.

M.L.: 1A, 1B, 3B.

## Disclosures

**Ethical Compliance Statement:** Ethical review and informed patient consent were not necessary for this work. We confirm that we have read the Journal's position on issues involved in ethical publication and affirm that this work is consistent with those guidelines.

**Funding Sources and Conflict of Interest:** No specific funding was received for this work. LGR reports a relationship with Newronika that includes: travel reimbursement. ML reports a relationship with Newronika that includes: equity or stocks. IUI reports a relationship with Newronika that includes: equity or stocks. FC reports a relationship with Newronika that includes: equity or stocks.

**Financial Disclosures for the Previous 12 Months:** The authors declare that there are no additional disclosures to report.

## Data Availability

Data sharing not applicable to this article as no datasets were generated or analysed during the current study.
